# Angiotensin receptor blocker ameliorates the development of tubulointerstitial injury and fibrosis associated with tubular proteinuria observed in subclinical diabetic kidney disease

**DOI:** 10.3389/fphar.2026.1871708

**Published:** 2026-08-20

**Authors:** Mariana R. Campos, Pedro Henrique S. R. Seabra, Laura B. F. Oliveira, Vitória L. Lacerda, Tawanne M. Souza, Mariana C. Moraes, Letícia Cristine C. dos Santos, Paula P. Campos, Celso Caruso-Neves, Diogo B. Peruchetti

**Affiliations:** 1 Department of Physiology and Biophysics, Institute of Biological Sciences, Federal University of Minas Gerais, Belo Horizonte, Brazil; 2 Department of Pharmacology, Institute of Biological Sciences, Federal University of Minas Gerais, Belo Horizonte, Brazil; 3 Department of General Pathology, Institute of Biological Sciences, Federal University of Minas Gerais, Belo Horizonte, Brazil; 4 Carlos Chagas Filho Biophysics Institute, Federal University of Rio de Janeiro, Rio de Janeiro, Brazil; 5 National Institute of Advanced Technologies in Diagnosis and Prognosis of Chronic and Neglected Diseases, INATEC, Rio de Janeiro, Brazil; 6 National Institute of Science and Technology in Nanobiopharmaceutics, INCT-Nanobiofar, Belo Horizonte, Brazil

**Keywords:** angiotensin receptor blocker (ARB), hyperglycemia, protein reabsorption, proteinuria, proximal tubule, renin-angiotensin system, subclinical diabetic kidney disease

## Abstract

**Introduction:**

Subclinical Diabetic Kidney Disease (_sub_DKD) is a clinically silent stage of kidney disease secondary to diabetes *mellitus* (DM), being a public health problem worldwide. The pathogenic mechanism involves hyperglycemia-induced tubulointerstitial injury associated with dysfunction of tubular protein reabsorption and proteinuria, even without any clinical signs like impaired glomerular function. Interestingly, angiotensin receptor blockers (ARBs), known as type 1 angiotensin II receptor (AT_1_R) antagonists, are effective against proteinuria in many kidney diseases, suggesting the potential involvement of the Renin-Angiotensin System. Herein, we studied the potential renoprotective effects of ARB on the pathogenesis of _sub_DKD.

**Methods:**

For that, we developed streptozotocin (STZ)-induced type 1 DM [with normal levels of glomerular flow rate] in male Wistar rats, which were daily treated with 30 mg/kg/day losartan, a well-known ARB, via gavage for 6 consecutive weeks starting after induction of diabetes. Four experimental groups were generated: 1) CONT, normoglycemic rat; 2) ARB, normoglycemic rat treated with losartan; 3) _sub_DKD, diabetic rat; 4) _sub_DKD+ARB, diabetic rat treated with losartan.

**Results:**

Comparing with the CONT group, the _sub_DKD group presented: 1) impaired tubular reabsorption of Na+ and Cl-; 2) proteinuria associated with dysfunction in cortical albumin endocytosis; 3) increased levels of urinary lactate dehydrogenase and γ-glutamyltransferase, markers of kidney and proximal tubule epithelial cell injuries, respectively; 4) increased kidney injury score and tubulointerstitial injury; 5) increased cortical type 1 collagen deposition. Interestingly, the ARB treatment ameliorated all parameters observed in diabetic rats (_sub_DKD+ARB group). No significant changes were observed in the ARB group.

**Discussion:**

Altogether, our findings indicated that ARB promotes renoprotective effects by attenuating AT1R-associated tubular damage and proteinuria of tubular origin observed in subDKD.

## 1 Introduction

Diabetic Kidney Disease (DKD) is a progressive kidney disease secondary to diabetes *mellitus* that is a public health problem worldwide (Ma et al., 2025; Tuttle et al., 2025). The pathophysiology of DKD is complex. During its early phase, there is a clinically silent stage of DKD (before early signs of glomerular hyperfiltration) called subclinical DKD (_sub_DKD) that is characterized by hyperglycemia, polyuria, glycosuria, and proteinuria associated with major tubulointerstitial injury rather than glomerular injury (Gilbert, 2017; Succar et al., 2017; Farias et al., 2023; Taylor et al., 2023). The deleterious effects of sodium/glucose cotransporter (SGLT)-mediated high glucose influx on proximal tubular epithelial cells (PTECs) are the central step in the _sub_DKD onset (Zhang et al., 2015; Gilbert, 2017; Johansen and Larsen, 2025; Martinez Leon et al., 2026). Previously, it was shown that, during _sub_DKD, the dysfunction of the PTEC endocytic machinery involved in albumin reabsorption mediates the development of tubulointerstitial injury and tubular albuminuria induced by hyperglycemia (Farias et al., 2023). Despite advances in this field, the mechanisms underlying these processes remain poorly understood.

One possible candidate is the octapeptide angiotensin II (Ang II) that belongs to the classical axis of the Renin-Angiotensin System (RAS) (Ferrario et al., 2022). The systemic and local Ang II levels are a result of the balance between its generation through angiotensin I hydrolysis promoted by angiotensin-converting enzyme (ACE) and its metabolism into angiotensin-(1–7) through angiotensin-converting enzyme 2 (ACE2) activity (Roman et al., 2016; Urushihara and Kobori, 2017; Ferrario et al., 2022). It has been shown that Ang II levels in the kidneys are increased under diabetic and proteinuric conditions (He et al., 2010; Peruchetti et al., 2021; Afonso et al., 2024; Peixoto et al., 2026). Interestingly, in both *in vitro* and *in vivo* studies, it has been shown that the activation of the Ang II/type 1 Ang II receptor (AT_1_R) pathway in PTECs mediates the dysfunction of PTEC endocytic machinery, consequently, the development of tubular proteinuria (Peruchetti et al., 2021; Afonso et al., 2024). Furthermore, it has been shown that the use of angiotensin receptor blockers (ARBs), also known as AT_1_R antagonists, attenuates glomerular and tubular injuries in established DKD (chronic phase) secondary to type 1 or type 2 diabetes *mellitus* (Habibi et al., 2019; Koszegi et al., 2019; Zhao et al., 2024). However, whether AT_1_R-mediated effects underlie the PTECs dysfunction as well as development of tubulointerstitial injury and fibrosis in _sub_DKD is still an open matter.

In this present study, we aimed to investigate the potential renoprotective effect of losartan, an ARB, on the development of tubulointerstitial injury and fibrosis, as well as tubular proteinuria in _sub_DKD. For that, we used the streptozotocin (STZ)-induced type 1 diabetes *mellitus* animal model (with no significant changes in glomerular function) and daily treated with 30 mg/kg/day losartan (via gavage) for 6 consecutive weeks after 2 weeks of type 1 diabetes induction. Our main findings showed that ARB treatment, in _sub_DKD, ameliorated tubular dysfunction and the development of tubulointerstitial injury and fibrosis associated with tubular proteinuria. These observations help us expand the current understanding of the role of RAS in DKD pathogenesis.

## 2 Methods

### 2.1 Animals and experimental design

Male Wistar rats (10 weeks old) were obtained from the Central Animal Facility of Federal University of Minas Gerais (UFMG). The animals were housed in an institutional experimental animal facility [Biotério do Instituto de Ciências Biológicas (BICBIO-2)] with free access to filtered water and standard chow, under controlled temperature and humidity conditions and a 12:12 h light-dark cycle. All animal care and experimental procedures were conducted in accordance with the NIH Guide for the Care and Use of Laboratory Animals and approved by the Institutional Ethical Committee (CEUA-UFMG#29/2023).

The total experimental period was 8 weeks. The rats were randomly divided into two major experimental groups: 1) normoglycemic rats, where rats received a single intraperitoneal injection with vehicle (citrate buffer, pH 4.5); and 2) diabetic rats, where rats received a single intraperitoneal injection of STZ (60 mg/kg). It is worth mentioning that, at 48 h after STZ injection, fasting blood glucose levels were assessed by collecting blood samples from the tail and measuring glucose concentrations using a glucometer (On Call Plus II®). Rats with blood glucose levels below 250 mg/dL were excluded from the study. In the 2^nd^ week of the experimental period, each experimental group was divided into two sub-groups: 1) that received filtered water as vehicle via gavage; 2) that received daily doses of 30 mg/kg/day losartan [a well-known angiotensin receptor blocker (ARB) and AT_1_R antagonist] diluted in filtered water via gavage for the remaining 6 consecutive weeks. The dosage of losartan was chosen based on previous studies (Landgraf et al., 2011; Silva-Filho et al., 2013; Silva et al., 2018; Peruchetti et al., 2021). In this way, four experimental groups were generated: 1) CONT group, normoglycemic rats - control group; 2) ARB group, normoglycemic rats daily treated with losartan 30 mg/kg/day for 6 consecutive weeks via gavage; 3) _sub_DKD group, STZ-induced diabetic rats; 4) _sub_DKD + ARB group, diabetic rats treated with losartan.

At the end of the experiment (8^th^ week), the rats were placed in metabolic cages for water intake measurements and 24 h urine collection. When indicated, the rats received a single intraperitoneal injection of BSA-FITC (5 μg/g, using saline as the vehicle) for posterior assessment of *in vivo* albumin uptake by kidneys (detailed in Section 2.3). After 30 min, the rats were euthanized under deep anesthesia by a single intraperitoneal injection of ketamine (100 mg/kg) and xylazine (10 mg/kg), followed by exsanguination. Blood was collected from the inferior vena cava using heparinized syringes and tubes, and plasma was obtained after centrifugation. Urine and plasma samples were used for biochemical analysis and assessment of renal function. Then, the kidneys were perfused with heparinized saline and processed for an *in vivo* albumin uptake assay as well as histological and histomorphometric analyses.

### 2.2 Assessment of renal function and kidney injury markers

Renal function was assessed as previously described (Peruchetti et al., 2019; Farias et al., 2023; Moraes et al., 2025). Briefly, the mice were housed in metabolic cages for a total of 72 h, with the first 48 h used for acclimation and the final 24 h for urine collection. To remove sediments, 24 h urine samples were clarified by centrifugation at 8,000 × g at room temperature for 10 min, and the supernatant was collected (the procedure was repeated 5 times). Clarified urine and plasma samples were used to measure creatinine, Na^+^, Cl^−^, glucose, and total protein levels using commercially available colorimetric or enzymatic kits (cat. #35, #124, #115, #84, and #36, respectively; Labtest, Lagoa Santa, MG, Brazil), according to the manufacturer’s instructions. These parameters were then used to calculate the renal clearance of each analyte (C_x_) and the fractional excretion of each analyte (FE_x_), as previously described (Peruchetti et al., 2019; Farias et al., 2023).

To assess kidney injury, urinary levels of lactate dehydrogenase (LDH, a marker of renal cell injury) and γ-glutamyl transferase (γ-GT, a specific marker of PTEC injury) were measured in clarified urine samples using enzymatic kits (cat. #86 and #105, respectively; Labtest). In addition, protein excretion mass (proteinuria, mg/24 h) was used as a marker of progressive kidney disease.

### 2.3 The assessment of *in vivo* albumin uptake

The *in vivo* albumin uptake assay was carried out as previously described (Peruchetti et al., 2021; Silva-Aguiar et al., 2022; Farias et al., 2023). Briefly, the rats were anesthetized and received a single intraperitoneal injection of 5.0 μg/g BSA-FITC. After 30 min, the kidneys were perfused with heparinized saline solution, followed by renal cortex isolation and homogenization (using a glass/teflon Potter Elvehjem homogenizer) in ice-cold Ringer’s solution [12.4 mM HEPES–Tris (pH 7.4), 140 mM NaCl, 2.7 mM KCl, 1.8 mM CaCl_2_, 1 mM MgCl_2_, 5 mM D-(+)glucose] supplemented with 1 mM phenylmethanesulfonylfluoride and protease inhibitor cocktail 1×. After being clarified twice by centrifugation at 1,000×g for 10 min, the intensity signals of organ-associated fluorescence (excitation = 480 nm, emission = 520 nm) were determined using a spectrofluorometer (Synergy HT, Biotek, Agilent Technologies, Santa Clara, CA, United States). Specific fluorescence was calculated as previously described (Farias et al., 2023).

### 2.4 Measurement of albumin uptake in renal cortex explants

Albumin uptake in renal cortex explants was measured as previously described (Silva-Aguiar et al., 2022). Briefly, perfused rat kidneys were used to extract renal cortex explants using a manual micrometer and placed in 24-well plates containing low-glucose DMEM (Gibco) at 37 °C and 5% CO_2_ supplemented with 1% antibiotics/antimycotics (Gibco) for 10 min (for equilibration) (Gómez-Jaramillo et al., 2022; Silva-Aguiar et al., 2022). Then, the explants have been incubated in different experimental conditions for 24 h: 1) normal glucose (NG, 5 mM, used as control); 2) high glucose (HG, 25 mM); 3) NG + losartan (LOS, 10^−7^ M); and 4) HG + LOS.

After treatment, the explants were washed out three times using Ringer’s solution at 37 °C, and then incubated with 100 μg/mL BSA-FITC for 30 min at 37 °C. After the reaction, the explants were extensively washed (11 times) with ice-cold Ringer solution to remove the unbound BSA-FITC. Then, the explants were homogenized, using a glass/teflon Potter Elvehjem homogenizer, in ice-cold lysis buffer [250 mm sucrose, 10 mm HEPES–Tris (pH 7.4), 2 mm EDTA, 5 mm phenylmethanesulfonyl fluoride, and protease inhibitor mixture (Sigma-Aldrich)]. The organ-associated fluorescence was determined using a spectrofluorometer (Synergy HT, Biotek, Agilent Technologies, Santa Clara, CA, United States). The fluorescence intensity was normalized by the total protein in the respective samples. The total protein measurement was carried out using the Folin phenol method (Lowry et al., 1951). Data is expressed as arbitrary units.

### 2.5 Histology and histomorphometric analysis

Histology and histomorphometric analyses were performed as previously described (Farias et al., 2023; Peres et al., 2023; Moraes et al., 2025). Briefly, kidney samples were fixed in 4% paraformaldehyde, embedded in paraffin, and sectioned at 5 µm thickness. Sections were stained with: 1) hematoxilin-eosin (HE) for kidney pathology score; 2) periodic acid-Schiff (PAS) for the evaluation of glomerular and tubular structural parameters; or 3) SiriusRed (SR, Direct Red 80, catalog no. 365548, Sigma-Aldrich, St. Louis, MO, United States) for the assessment of collagen deposition. One section per kidney was analyzed. Thirty images per section were acquired using systematic random sampling with a Zeiss microscope (AxioCam M2, Carl Zeiss, Jena, Germany). Quantitative analyses were performed using ImageJ software (National Institutes of Health, Bethesda, MD, United States) and Image Pro-Plus version 4.5.0.29 (Media Cybernetics, Rockville, MD, United States).

In HE-stained sections, the acquired images were used by the renal histopathologists (LCCS and PPC) to determine the kidney injury score using previously published protocols (Hoshino et al., 2015; Qi et al., 2017; Toprak et al., 2020). Briefly, the parameters assessed were: glomerular lesions, interstitial inflammation, necrosis, and hydropic (vacuolar) degeneration. Each parameter analyzed was classified according to the following scale: 0 to absent, 1 to mild, 2 to moderate, and 3 to severe alterations. The sum of points obtained from all parameters analyzed is the final score value (related to a specific animal).

In PAS-stained sections, the following glomerular parameters were evaluated: glomerular cellularity (number of cell nuclei within the glomerular tuft normalized to tuft area); mesangial matrix expansion (PAS-positive areas normalized to tuft area); and glomerular tuft area (normalized to total glomerular area). Tubular parameters included the number of interstitial cells and of PTECs.

In SiriusRed-stained sections, collagen deposition was quantified by measuring the density of red-stained fibers and normalized to the total tissue area. Collagen accumulation was calculated using the following formula: collagen deposition (%) = (red-stained area/total tissue area) x 100. Perivascular areas were excluded from the analysis to ensure methodological accuracy. In addition, type 1 collagen expression was analyzed by a light polarized microscope (Nikon Eclipse 80i, Nikon Instruments Inc., Melville, NY, United States). All image acquisition and histomorphometric analyses were performed in a blinded manner.

### 2.6 Statistical analysis

All graphs and statistical analyses were performed using GraphPad Prism software version 8.0.2 (GraphPad Software, Inc., San Diego, CA, United States). The normal distribution was analyzed using the Shapiro-Wilk test. Normally distributed data were expressed as mean ± SD, while non-normally distributed data were expressed as median with interquartile range. In the graphs, symbols represent the number of independent experiments (n). Comparisons between groups were performed using a two-way ANOVA test followed by Tukey’s multiple comparisons test for normally distributed data or the Kruskal–Wallis test followed by Dunn’s multiple comparisons test for non-normally distributed data. Statistical significance was defined as P < 0.05. Correlation analyses were performed using Pearson correlation test for Gaussian distributions and Spearman correlation test for non-Gaussian distributions.

## 3 Results

### 3.1 Role of Ang II/AT_1_R axis in the glomerular and tubular function during _sub_DKD

Initially, we assessed the potential role of Ang II/AT_1_R axis blockade in the glomerular and tubular function in _sub_DKD. For that, we generated four experimental groups (detailed in the Methods section): 1) CONT group, normoglycemic rats - control group; 2) ARB group, normoglycemic rats, daily treated with losartan 30 mg/kg/day for 6 consecutive weeks via gavage; 3) _sub_DKD group, STZ-induced diabetic rats; 4) _sub_DKD + ARB group, diabetic rats treated with losartan. The Table 1 shows the basic clinical parameters of the experimental groups. No significant changes were observed in the levels of plasma Na^+^, plasma Cl^−^, or plasma proteins in any experimental groups (P > 0.05). We observed a significant decrease in body weight by 31% (P < 0.0001) along with a significant increase in KW/BW (1.80-fold; P = 0.018), food intake (1.72-fold; P = 0.0016), water intake (4.30-fold; P < 0.0001), urinary volume (9.4-fold; P < 0.0001), glycemia (3.1-fold; P < 0.0001), and glycosuria (840.0-fold; P < 0.0001) in _sub_DKD when compared with the CONT group. The ARB treatment did not change these effects induced by hyperglycemia (_sub_DKD + ARB group). In addition, no changes were observed with ARB treatment alone (ARB group).

**TABLE 1:** Clinical parameters.

Parameters	CONT (n = 9)	ARB (n = 9)	_sub_DKD (n = 9)	_sub_DKD + ARB (n = 10)
Body weight (g)	402.1 ± 18.8	386.6 ± 17.3	278.3 ± 52.1*	229.9 ± 43.7*
KW/BW (10^−3^)	3.17 ± 0.24	3.47 ± 0.40	5.71 ± 2.46*	6.14 ± 0.81*
Food intake (g/day)	21.50 ± 4.12	20.56 ± 6.38	37.00 ± 5.74*	31.86 ± 8.80*
Water intake (mL/day)	30.11 ± 9.00	26.56 ± 4.93	129.40 ± 32.09*	122.00 ± 22.17*
Urinary volume (mL/day)	12.21 ± 5.34	10.71 ± 2.35	114.90 ± 17.78*	114.10 ± 22.89*
Glycemia (mg/dL)	157.00 ± 18.64	143.40 ± 38.81	486.10 ± 96.10*	593.00 ± 68.96*
Glycosuria (mg/dL)	7.5 ± 4.6	140.3 ± 125.8	6,351.0 ± 2,135.0*	6,910.0 ± 1,632.0*
Plasma Na^+^ (mmol/L)	146.00 ± 12.06	145.00 ± 12.82	156.90 ± 11.60	150.70 ± 5.29
Plasma Cl^−^ (mEq/L)	124.4 ± 9.08	138.5 ± 10.42	127.3 ± 27.85	116.3 ± 14.70
Plasma proteins (g/dL)	6.38 ± 1.20	8.48 ± 1.50	7.17 ± 2.62	7.33 ± 2.85

Data are expressed as mean ± standard deviation (SD). KW/BW, kidney weight: body weight ratio. *P < 0.05 versus CONT, group.

**FIGURE 1:**
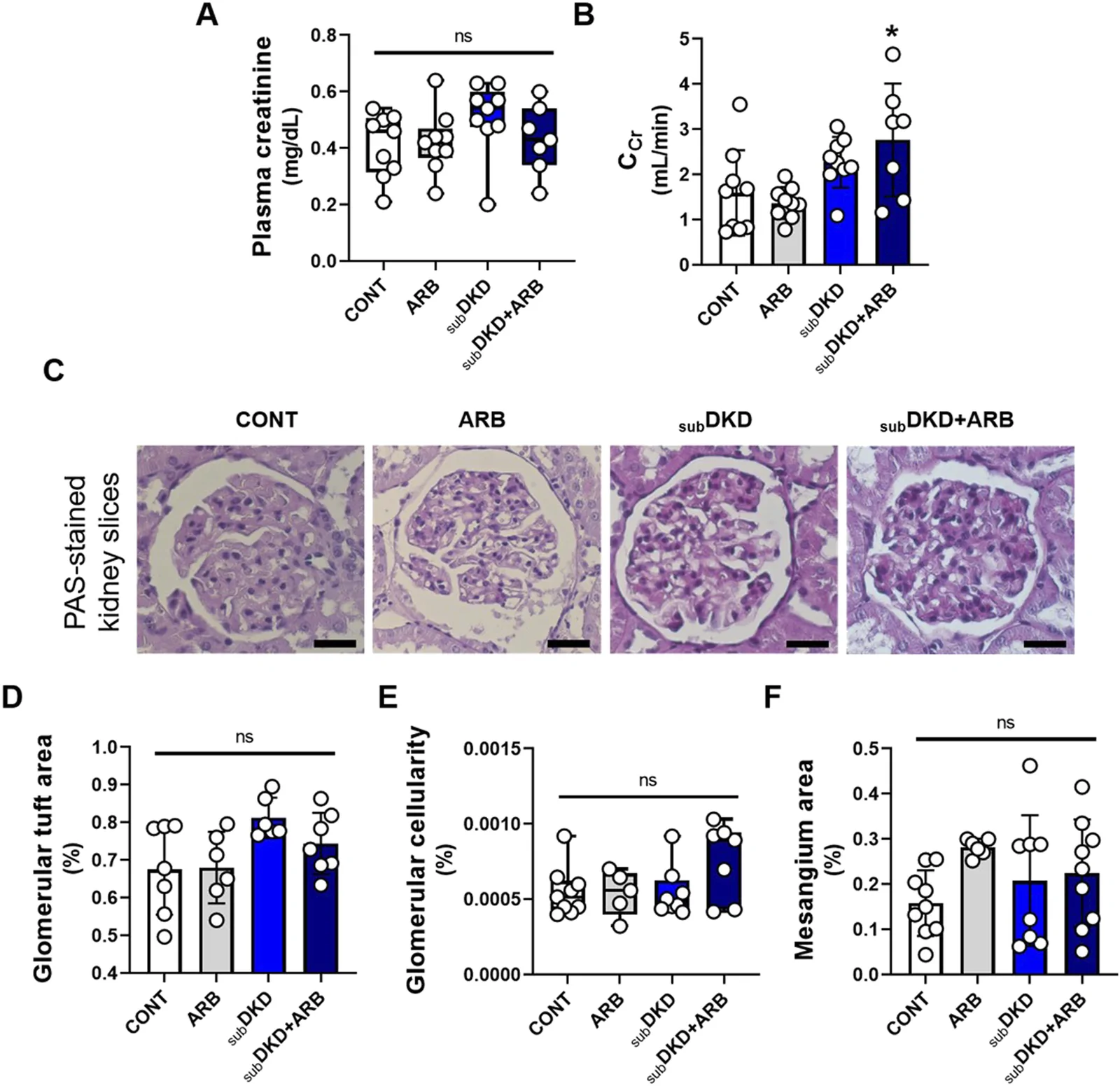
Glomerular structure and function are preserved in subclinical diabetic kidney disease. Male Wistar rats (10 weeks old) were used to develop streptozotocin-induced type 1 diabetes mellitus, and then, when indicated, daily treated with losartan (30 mg/kg/day) via gavage during 8 consecutive weeks. Four groups were generated (as described in the Methods section): 1) CONT group (n = 9); 2) ARB group (n = 9); 3) _sub_DKD group (n = 9); and 3) _sub_DKD + ARB group (n = 7). **(A)** Plasma creatinine concentration, **(B)** creatinine clearance (C_Cr_), **(C)** Representative images of micrographs from kidney cortex slices stained with Periodic acid-Schiff (PAS) reagent (scale bar = 50 μm). The glomeruli analyzed are shown in the inset presented in the lower panels. Based on these images, the glomerular tuft area **(D)** the glomerular cellularity **(E)** and the mesangium area **(F)** were measured. Open circles represent individual animals. Normally distributed data were expressed as mean ± SD, while non-normally distributed data were expressed as median with interquartile range. Statistical analysis for panels **(B,D,F)** was performed using a two-way ANOVA test following Tukey’s post-test. Statistical analysis for panels **(A,E)** was performed using the Kruskal–Wallis test following Dunn’s post-test. n. s., not significant. *P < 0.05 versus CONT group.

Figure 1 shows the assessment of glomerular function and structure parameters. We observed that no significant changes in plasma creatinine (P > 0.05; Figure 1A). The creatinine clearance (C_Cr_, a well-known marker of glomerular filtration rate) was increased only in the _sub_DKD + ARB group (Figure 1B; 1.74-fold; P = 0.037). In agreement with those findings, we also did not observe any significant changes in glomerular tuft area, glomerular cellularity, and mesangial matrix density among all experimental groups (P > 0.05; Figures 1C–F).

**FIGURE 2:**
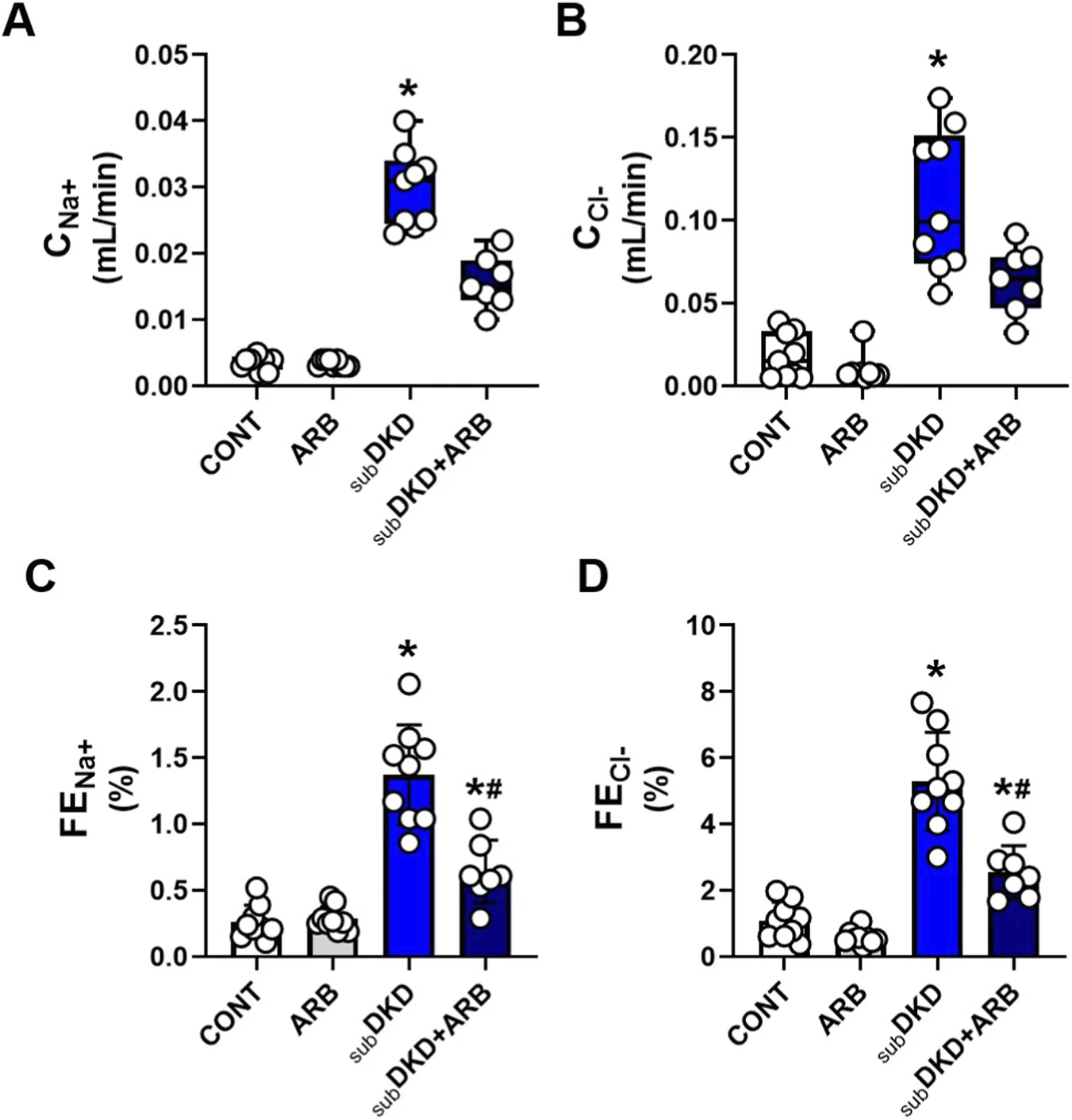
Losartan treatment attenuated the tubular dysfunction observed in subclinical diabetic kidney disease. Male Wistar rats (10 weeks old) were used to develop streptozotocin-induced type 1 diabetes mellitus, and then, when indicated, daily treated with losartan (30 mg/kg/day) via gavage during 8 consecutive weeks. Four groups were generated (as described in the Methods section): 1) CONT group (n = 9); 2) ARB group (n = 9); 3) _sub_DKD group (n = 9); and 3) _sub_DKD + ARB group (n = 7). **(A)** Renal clearance of Na^+^ (C_Na+_), **(B)** renal clearance of Cl^−^ (C_Cl-_), **(C)** fractional excretion of Na^+^ (FE_Na+_), and **(D)** fractional excretion of Cl^−^ (FE_Cl-_). Open circles represent individual animals. Normally distributed data were expressed as mean ± SD, while non-normally distributed data were expressed as median with interquartile range. Statistical analysis for panels **(A,B)** was performed using the Kruskal–Wallis test following Dunn’s post-test. Statistical analysis for panels **(C,D)** was performed using a two-way ANOVA test following Tukey’s post-test. *P < 0.05 versus CONT group. #P < 0.05 versus the _sub_DKD group.

Next, we assessed the potential role of AT_1_R inhibition on tubular function in _sub_DKD. We only observed a significant increase in both levels of C_Na+_ (8.37-fold; P = 0.0001) and C_Cl-_ (6.17-fold; P = 0.0007) in the _sub_DKD group (Figures 2A,B). To verify if these high urinary excretion of Na^+^ and Cl^−^ are due to tubular dysfunction in _sub_DKD, we assessed the levels of FE_Na+_ and FE_Cl-_. We observed a significant increase in FE_Na+_ (5.23-fold; P < 0.0001) and FE_Cl-_ (4.82-fold; P < 0.0001) in the _sub_DKD group when compared to CONT group (Figures 2C,D). Interestingly, we observed that ARB treatment attenuated these effects (_sub_DKD + ARB group), indicating that ARB treatment abolished the tubular dysfunction observed in _sub_DKD. It is worth mentioning that none of these parameters assessed were changed in the ARB group (Figures 2A–D).

### 3.2 Ang II/AT_1_R axis mediates the development of tubular proteinuria in _sub_DKD

**FIGURE 3:**
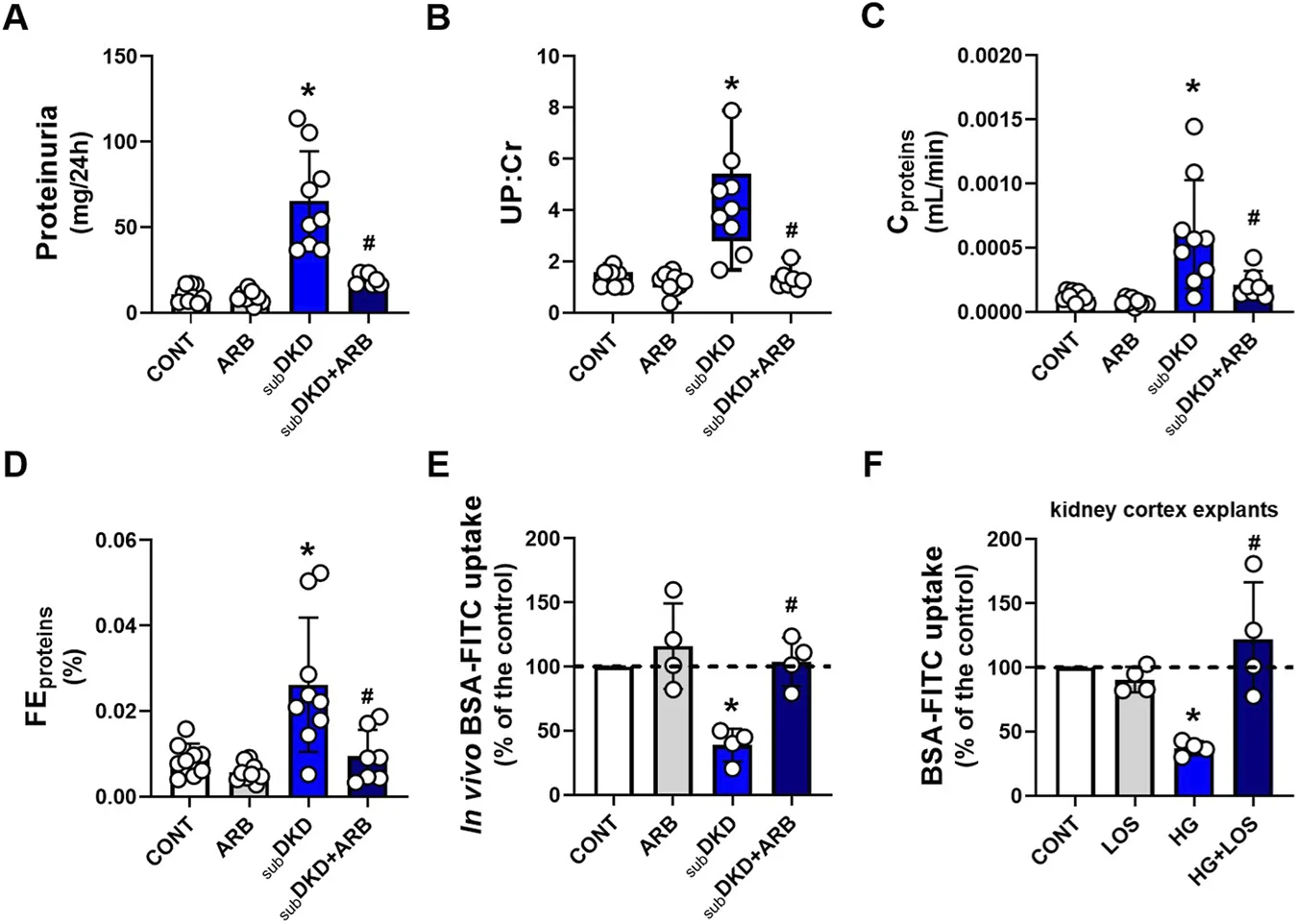
Losartan treatment ameliorated the development of tubular proteinuria observed in subclinical diabetic kidney disease. Male Wistar rats (10 weeks old) were used to develop streptozotocin-induced type 1 diabetes mellitus, and then, when indicated, daily treated with losartan (30 mg/kg/day) via gavage during 8 consecutive weeks. Four groups were generated (as described in the Methods section): 1) CONT group (n = 9); 2) ARB group (n = 9); 3) _sub_DKD group (n = 9); and 3) _sub_DKD + ARB group (n = 7). **(A)** Urinary protein mass excretion (mg/24 h), **(B)** urinary protein:creatinine ratio (UP:Cr), **(C)** renal clearance of proteins (C_proteins_), and **(D)** fractional excretion of proteins (FE_proteins_). **(E)** *In vivo* BSA-FITC uptake assay (n = 4/group). **(F)** *Ex vivo* assay showing the direct effect of high glucose (D (+)-glucose 25 mM) and 10^−7^ M losartan treatment on the BSA-FITC uptake in renal cortex explants (n = 4). Open circles represent individual animals. Normally distributed data were expressed as mean ± SD, while non-normally distributed data were expressed as median with interquartile range. Statistical analysis for panels **(A,C–F)** was performed using a two-way ANOVA test following Tukey’s post-test. Statistical analysis for panel **(B)** was performed using the Kruskal–Wallis test following Dunn’s post-test. *P < 0.05 versus CONT group. #P < 0.05 versus the _sub_DKD group.

Initially, we decided to investigate the effect of losartan on the development of proteinuria observed in _sub_DKD. We observed that the levels of proteinuria (6.0-fold; P < 0.0001), urinary protein:creatinine ratio (UP:Cr, 3.26-fold; P = 0.0045), and C_proteins_ (5.17-fold; P = 0.0004) only increased in the _sub_DKD group (Figures 3A–C). Since we did not observe any changes in C_Cr_, we investigated possible changes in FE_proteins_, an indicator of tubular protein reabsorption. We observed a significant increase in FE_proteins_ (3.01-fold; P = 0.0012) in the _sub_DKD group when compared with the CONT group (Figure 3D). Interestingly, the daily treatment with ARB abolished this effect observed in early DKD (_sub_DKD + ARB group). No significant changes were observed in the ARB group.

To confirm the protective role of losartan on tubular proteinuria, we assessed the *in vivo* BSA-FITC uptake, a marker of albumin endocytosis in PTECs. We observed that *in vivo* BSA-FITC uptake was reduced by 61% (P = 0.0051) in the _sub_DKD group compared with the CONT group (Figure 3E). Interestingly, losartan treatment completely blocked this inhibitory effect in diabetic rats (as observed in the _sub_DKD + ARB group). Importantly, no changes were observed in the ARB group. Furthermore, to verify the direct influence of high glucose (HG) influx in PTECs and renal AT_1_R on these processes, we used 1-mm rat kidney explants cultured with HG (25 mM) and/or 10^−7^ M losartan for 24 h to assess the BSA-FITC uptake. We observed that HG significantly inhibited the BSA-FITC uptake by 63% (P = 0.01), and the co-incubation of explants with HG and losartan abolished the inhibitory effect of HG on BSA-FITC uptake (Figure 3F).

### 3.3 Ang II/AT_1_R axis mediates the development of tubulointerstitial injury and fibrosis in _sub_DKD

**FIGURE 4:**
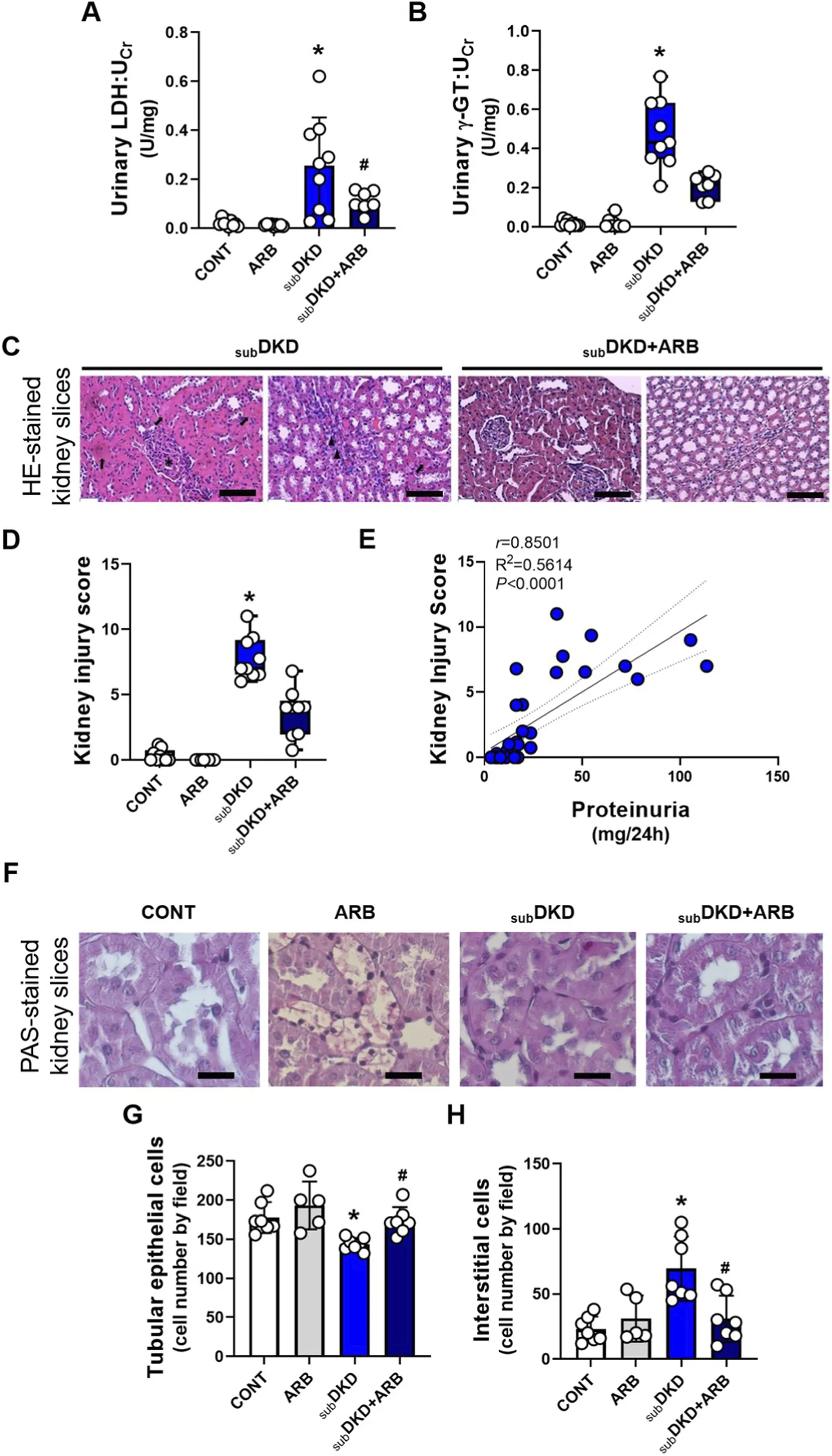
Losartan treatment attenuated the development of tubulointerstitial injury observed in subclinical diabetic kidney disease. Male Wistar rats (10 weeks old) were used to develop streptozotocin-induced type 1 diabetes mellitus, and then, when indicated, daily treated with losartan (30 mg/kg/day) via gavage during 8 consecutive weeks. Four groups were generated (as described in the Methods section): 1) CONT group (n = 9); 2) ARB group (n = 9); 3) _sub_DKD group (n = 9); and 3) _sub_DKD + ARB group (n = 7). Urinary lactate dehydrogenase (LDH, **(A)** and urinary γ-glutamyltransferase (γ-GT, **(B)** were measured. Open circles represent individual animals. Normally distributed data were expressed as mean ± SD, while non-normally distributed data were expressed as median with interquartile range. **(C)** Representative images from kidney slices stained with HE (scale bar = 50 μm). Based on these images, the kidney injury score (**(D)** n = 5–7) was measured. **(E)** Positive Spearman correlation between the kidney injury score and proteinuria. **(F)** Representative images of micrographs from kidney cortex slices stained with Periodic acid-Schiff (PAS) reagent (scale bar = 50 μm). Based on these images, the number of tubular epithelial cells (**(G)** n = 5–7) and the number of interstitial cells (**(H)** n = 5–7) were measured. Statistical analysis for **(A,G,H)** was performed using a two-way ANOVA test following Tukey’s post-test. Statistical analysis for panels **(B,D)** was performed using the Kruskal–Wallis test following Dunn’s post-test. *P < 0.05 versus CONT group. #P < 0.05 versus the _sub_DKD group.

Initially, we investigated the potential impact of AT_1_R antagonism on the development of tubulointerstitial injury and fibrosis in _sub_DKD. In Figures 4A,B, we observed an increased urinary LDH (11.9-fold; P = 0.0002) and urinary γ-GT (29.4-fold; P = 0.0005) in the _sub_DKD group when compared to the CONT group. Interestingly, the treatment of diabetic rats with losartan attenuated these modifications (_sub_DKD + ARB group). No changes were observed when the animals were treated with ARB alone (ARB group).

To confirm that these alterations in urinary kidney injury markers correlate with tubular structural changes, initially, we assessed the HE-stained kidney slices to measure the kidney injury score (Figures 4C,D). We observed that only the _sub_DKD presented a significant increase in kidney injury score (24.4-fold; P = 0.0002), indicating a renoprotective effect of ARB (Figure 4D). In addition, we found a positive Spearman’s correlation between kidney injury score and proteinuria (Figure 4E; *r* = 0.8501, r^2^ = 0.5614, P < 0.0001).

For the histomorphometric analyses, we used PAS-stained kidney slices to measure the number of tubular epithelial cells and interstitial cells. We observed that the _sub_DKD group presented a reduced number of tubular epithelial cells by 19% (P = 0.0168) and an increased number of interstitial cells (3.04-fold; P = 0.0005) when compared to the CONT group (Figures 4F–H). These effects were attenuated by ARB treatment (_sub_DKD + ARB group). No changes were observed in the ARB group.

**FIGURE 5:**
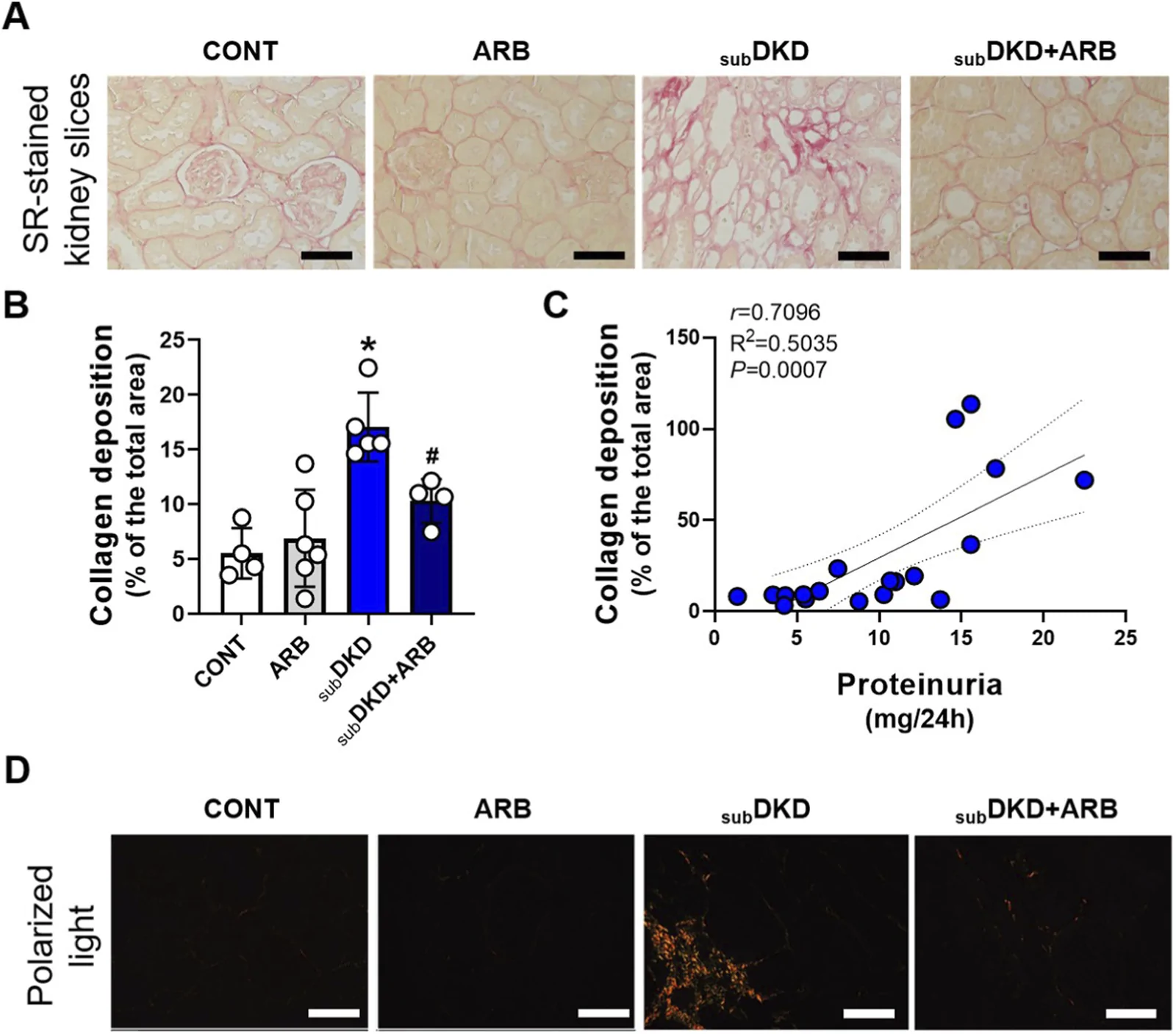
Losartan treatment attenuated the development of fibrosis observed in subclinical diabetic kidney disease. Male Wistar rats (10 weeks old) were used to develop streptozotocin-induced type 1 diabetes mellitus, and then, when indicated, daily treated with losartan (30 mg/kg/day) via gavage during 8 consecutive weeks. Four groups were generated (as described in the Methods section): 1) CONT group (n = 9); 2) ARB group (n = 9); 3) _sub_DKD group (n = 9); and 3) _sub_DKD + ARB group (n = 7). **(A)** Representative images of micrographs from kidney cortex slices stained with SiriusRed (scale bar = 50 μm). **(B)** Quantification of intensity related to red collagen fibers (n = 4–6). Open circles represent individual animals. Normally distributed data were expressed as mean ± SD. **(C)** Positive Pearson correlation between kidney injury score and proteinuria. **(D)** Type 1 collagen deposition observed in the polarized light microscopy. Statistical analysis for panel **(B)** was performed using a two-way ANOVA test following Tukey’s post-test. *P < 0.05 versus CONT group. #P < 0.05 versus the _sub_DKD group.

In Figure 5, we showed SiriusRed-stained kidney slices to assess the levels of collagen deposition. We observed that tubular collagen deposition was increased only in the _sub_DKD group (3.08-fold; P = 0.0006) when compared to CONT, _sub_DKD + ARB, and ARB groups (Figures 5A,B). In addition, a positive Pearson’s correlation was found between collagen deposition and proteinuria (Figure 5C; *r* = 0.7096, r^2^ = 0.5035, P = 0.0007). Interestingly, using polarized microscopy, we observed similar effects in type 1 collagen deposition across the experimental groups assessed (Figure 5D).

## 4 Discussion

Herein, our results showed that, in _sub_DKD, hyperglycemia was associated with increased HG influx in PTECs, impaired tubular reabsorption of electrolytes and proteins, and the development of tubular proteinuria in association with tubulointerstitial injury and fibrosis. Interestingly, the AT_1_R blockade ameliorated all these deleterious effects induced by hyperglycemia, indicating its renoprotective potential in the onset of DKD and expanding the current understanding of the role of angiotensin peptides in DKD pathogenesis.

In the present study, we used a _sub_DKD animal model in accordance with the tubulocentric view of DKD pathogenesis, where changes in PTECs injury and dysfunction are far more pronounced than changes in the glomerulus (Gilbert, 2017). Using electron transmission microscopy and/or electron scanning microscopy techniques, it has been shown that minor ultrastructural changes (including glomerular basement membrane and podocytes) have been reported at 8 weeks after STZ-induced diabetes (Moriya et al., 1993; Miko et al., 2016; Conti et al., 2018; Rooney et al., 2025). Despite these minor changes, the glomerular permeability for albumin and anionic ferritin as well as high-molecular weight proteins remains unchanged in this time-frame (Boyd et al., 1996; Russo et al., 2009; Farias et al., 2023). In addition, the expression of nephrin (key component of glomerular permeability) was not changed in subclinical kidney diseases such as _sub_DKD (Forbes et al., 2002; Lakshmanan et al., 2011; Peruchetti et al., 2020). These findings suggest that hyperglycemia may affect both glomerular and tubular compartments during early DKD, although tubular dysfunction appears to be more prominent at the stage examined in the present study.

ARB or ACE inhibitors (ACEi) showed renoprotective and anti-proteinuric effects in chronic kidney disease patients with diabetes and in animal models of established DKD (Lewis et al., 1993; Brenner et al., 2001; Andersen et al., 2003; Teles et al., 2009; Yao et al., 2018; Habibi et al., 2019; Koszegi et al., 2019; Blazek and Bakris, 2023; Zhao et al., 2024), where the disease was found in advanced stages (compromised both glomerular and tubular functions). Regarding the early stage of DKD, Tojo et al. (2003), using STZ-induced diabetic male Sprague-Dawley rats, showed that quinapril (an ACEi, 3 mg/kg/day) and candesartan (an ARB, 0.05 mg/kg/day) also presented renoprotective effects. However, within this time frame, the authors cannot determine whether the main effect is attributable to hyperglycemia or to the direct effects of STZ on PTECs. In this context, it is well-known that STZ promotes reversible AKI (Perry and Weiss, 1982; Lyrio et al., 2024). In this present study, we used STZ-induced diabetic Wistar rats, which were allowed to recover from the direct effects of STZ, and then started the daily ARB treatment only 2 weeks after the single STZ injection. Importantly, we followed up on these animals until the 8^th^ week after a single STZ injection. At this time-frame, we clearly observed the influence of hyperglycemia on the kidneys (ruling out the direct effect of STZ), still in the presence or absence of ARB treatment.

It was already shown that HG influx in PTECs inhibited the (Na^+^+K^+^)ATPase α1 subunit expression and enzyme activity, culminating in impaired tubular Na^+^ reabsorption and increased natriuresis observed in early DKD (Silva-Aguiar et al., 2023). However, the mechanism involved in this process still needs elucidation. Herein, we observed that ARB treatment attenuated the impairment of tubular Na^+^ and Cl^−^ reabsorption promoted by hyperglycemia, suggesting that AT_1_R mediates, at least in part, the tubular dysfunction observed in _sub_DKD. In agreement with this hypothesis, some studies have shown that high concentrations of Ang II through AT_1_R inhibited tubular Na^+^ reabsorption (Harris and Young, 1977; Han et al., 2000).

It is well-known that HG influx into PTECs promotes tubular proteinuria in association with the dysfunction of megalin-dependent albumin endocytosis in _sub_DKD (Tojo et al., 2001; Farias et al., 2023; Silva-Aguiar et al., 2023). Interestingly, previous *in vitro* and *in vivo* studies showed that Ang II via AT_1_R causes a reduction in albumin endocytosis and tubular proteinuria (Hosojima et al., 2009; Peruchetti et al., 2021). Regarding DKD, it was shown that: quinapril or telmisartan attenuated the impairment of megalin expression and proteinuria in 2 weeks after STZ injection (Tojo et al., 2003); and 2) HG-induced Ang II/AT_1_R pathway inhibited megalin-dependent albumin endocytosis in the LLC-PK1 cell line (Afonso et al., 2024). In the present study, we observed that ARB treatment attenuated: 1) the increased fractional excretion of proteins (marker of impaired tubular protein reabsorption) and tubular proteinuria during _sub_DKD; and 2) the impaired albumin uptake in HG-treated explants. These findings indicate that AT_1_R may mediate this process.

There is a correlation among tubular proteinuria, the dysfunction of tubular protein reabsorption, and the development of tubulointerstitial injury in different kidney diseases (Theilig et al., 2007; Grieco et al., 2018; Larsen et al., 2018; Peruchetti et al., 2020; 2021; Farias et al., 2023; Rodrigues et al., 2024; Moraes et al., 2025). In addition, evidence pointed out that megalin deficiency promotes tubular pro-inflammatory phenotype and damage (Long et al., 2022; Li et al., 2026). Herein, we observed that ARB treatment in _sub_DKD promoted: 1) improvement in both kidney and specific PTECs injury markers; 2) an increase in the number of PTECs and a reduction in the number of interstitial cells; and 3) restoration of tubular protein reabsorption. These findings suggest that AT_1_R-mediated dysfunction of tubular protein reabsorption may contribute to the development of tubulointerstitial injury in _sub_DKD.

A strict correlation between tubulointerstitial injury and kidney fibrosis has been observed (Gorriz and Martinez-Castelao, 2012; Cortinovis et al., 2024). It has been reported increased transforming growth factor-β (TGF-β, a known pro-fibrotic cytokine), collagen fibers, vimentin, and α-smooth muscle actin (α-SMA, a marker of mesenchymal cells and fibroblasts) expression in rat kidneys with established DKD (Xue et al., 2023; Wang et al., 2024). Higher collagen fiber accumulation is also observed in subclinical kidney disease (Teixeira et al., 2019; Peruchetti et al., 2020; Peres et al., 2023). Herein, we observed an increase in total tubular collagen deposition, specifically type 1 collagen fibers, in _sub_DKD. In agreement, it has been shown that HG increased: 1) TGF-β and its receptors mRNA expression in the LLC-PK1 cell line (Guh et al., 1996); 2) TGF-β-dependent Smad3 phosphorylation, fibronectin, type IV collagen, and α-SMA expression in HK-2 cell line (Ndibalema et al., 2020); 3) TGF-β and type 1 collagen expression in cultured murine renal fibroblasts (Han et al., 1999). Furthermore, an enhanced profibrotic microenvironment (containing injured PTECs with active fibroblasts) was observed in human DKD (Dumoulin et al., 2026).

It has been shown that activation of the Ang II/AT_1_R pathway mediates the increase in pro-fibrotic mediators and kidney fibrosis in established DKD (Wolf, 1998; Habibi et al., 2019; Koszegi et al., 2019; Zou et al., 2022). Our results showed that ARB oral treatment markedly attenuated these pro-fibrotic changes in _sub_DKD. Consistent with our findings, it has been shown that ARB treatment has been reported to attenuate PTEC-specific gene expression changes associated with pro-fibrotic responses in experimental DKD (Wu et al., 2022).

Previous studies showed increased intrarenal and urinary Ang II levels in 8 weeks after STZ injection (Moon et al., 2008; He et al., 2010). In agreement, it was observed: 1) increased intrarenal Ang II in subclinical kidney disease (Peixoto et al., 2026); 2) HG-induced Ang II secretion in PTEC lines (Visavadiya et al., 2011; Afonso et al., 2024); 3) increased *AGT* gene in diabetic kidneys (Wu et al., 2022); 4) increased renal ACE expression (Moon et al., 2008); and 5) reduced renal ACE2 expression (Lakshmanan et al., 2011). Furthermore, renal AT_1_R expression is also increased in 3 weeks after STZ injection (Harrison-Bernard et al., 2002; Lakshmanan et al., 2011). Together, these findings provides a plausible basis for an activated intrarenal Ang II/AT_1_R axis in _sub_DKD. However, because we did not directly assess RAS components in the present study, this hypothesis requires further investigation.

Collectively, these findings suggest a potential mechanistic link between AT_1_R-mediated PTEC dysfunction and tubulointerstitial injury. Megalin functions not only as the main receptor for tubular protein reabsorption but also as a signaling platform capable of modulating proinflammatory and profibrotic pathways (Caruso-Neves et al., 2006; Nielsen and Christensen, 2010; Peruchetti et al., 2014). Thus, impaired megalin-dependent protein reabsorption induced by hyperglycemia and/or Ang II/AT_1_R activation may promote the release of mediators that influence neighboring interstitial cells (Peruchetti et al., 2018; Torsello et al., 2023; Afonso et al., 2024; Xu et al., 2024). Recent single-cell and spatial transcriptomic studies support the importance of epithelial-interstitial crosstalk in DKD (Wu et al., 2022; Bai et al., 2024; Dumoulin et al., 2026); however, the specific mediators involved in this process during _sub_DKD remain unknown.

In conclusion, our findings indicate that tubular dysfunction, characterized by impaired electrolyte and protein reabsorption, is a prominent feature of _sub_DKD and is associated with tubulointerstitial injury and fibrosis despite the absence of overt changes in glomerular structure and function. Daily oral losartan treatment for 6 weeks attenuated these alterations, supporting a role for AT_1_R signaling in the early renal consequences of diabetes. Based on our findings and the existing literature, we propose that hyperglycemia-induced PTEC dysfunction may involve the activation of the intrarenal Ang II/AT_1_R axis, contributing to impaired tubular protein reabsorption and subsequent tubulointerstitial injury and fibrosis. These findings provide new insights into the potential role of AT_1_R signaling in the pathogenesis of early DKD. Further studies are required to directly determine the contribution of intrarenal RAS activation and epithelial-interstitial crosstalk to these processes during _sub_DKD.

## Data Availability

The raw data supporting the conclusions of this article will be made available by the authors, without undue reservation.
